# Finding a Good Balance between Pressure to Publish and Scientific Integrity and How to Overcome Temptation of Scientific Misconduct

**DOI:** 10.3390/tomography8040155

**Published:** 2022-07-19

**Authors:** Emilio Quaia, Federica Vernuccio

**Affiliations:** Department of Radiology, University of Padova, Via Giustiniani 2, 35128 Padova, Italy; federica.vernuccio@aopd.veneto.it

Nowadays, there is a progressive increase in pressure to publish as well as greater emphasis on publishing in high impact journals, even sometimes with significant financial incentives attached. Although publication has always been one of the driving forces of scholarly research, scientific publication has been transformed within a new system to score people working in the research system based on bibliometric indices [1]. Pressure to publish has now reached a new level of importance although not all the sides of this phenomenon are positive [1].

The motto “publish or perish” is very common in the academic world and its implications are not perceived in a particularly positive light by researchers. The uncontrolled pressure to publish could represent a pathway to scientific misconduct since metrics such as numbers of publications and citations have achieved the highest level of importance, with a consequent increase in personal scores and bibliometric indices which seem to have become more important than scientific rigour and methodology. Some psychologists consider publication pressure to be a form of psychological stress which can lead to diminished ethical decision making and risky behavior such as scientific misconduct [2].

Fabrication, falsification, and plagiarism or redundant publication and other forms of scientific misconduct such as selective reporting, intentional deletion or amplification of data, selective citing, and guest authorships represent the most common form of scientific misconduct [3] and they are frequently perceived as directly related to pressure to publish [3]. Nowadays, the motto “publish or perish” has overridden the dilemma of “publish and be ethical”, which represents the paramount issue in the academic world. In our opinion, there is a list of good practices which could help to achieve a good balance between understandable and unavoidable pressure to publish and scientific misconduct. They can be listed as follows:

1. Be consistent and constant in scientific writing. Never stop and be persistent with your manuscript. Dedicate at least 10–20 min every day to your manuscript to progressively improve your writing skills and the quality of your manuscript.
2. Try to publish narrative reviews, pictorial reviews, book chapters and editorials as well as original research articles. Since narrative reviews, pictorial reviews, and editorials and book chapters are both referenced on bibliometric database such as Scopus, all these types of publications can increase your bibliometric indices with the possibility to dedicate more time to a limited number of original papers.
3. Avoid the so-called “salami slicing” phenomenon. If you combine all relevant points that derive from your research into one single article providing multiple clinically relevant messages, your paper is more likely to be published in high-impact journals and receive more citations rather than if you were to divide the messages into multiple papers that are likely to be published in low-impact journals and then receive only a few citations per paper.
4. Try to limit your own scientific production to one or two scientific manuscripts as first author per year and target high impact factor journals especially in the first submission cycle. It is much better to publish a few manuscripts in high impact factor journals than many manuscripts with a lower impact and citation probability.
5. Actively participate in as many other studies and grants as possible without acting as the reference author and be collaborative and find accountability partners. Research collaborations, in the short term, will require a level of additional effort, but in the mid- and long- term they will likely result in the multiplication of good papers and better clinical collaboration.
  - Within your department:
    - If a research team in your area of expertise does not exist yet, you can send a “call-to-action” with details outlining the required time, effort and area of research activity to all your colleagues in order to create a team with people that share your interest in research. You then need to trust them, share your ideas, discuss and divide the different tasks equally within the group and provide deadlines by which to complete the work. Of note, mentoring others does not mean leaving them to work completely independently. It is highly important to organize periodical meetings to share the progress of results and see if there are any concerns that could affect the progression of activities.
    - If there are already research teams, let them know of your availability to participate even with small tasks at the very beginning, and ask them if they are willing to help you in your main research topics.
  - Within your hospital, if you know any research teams in other departments who work on topics you are interested in, involve them actively in your projects, even with small tasks, and provide them with your availability to perform small tasks in their projects.
  - You may involve research teams from other hospitals who are interested in your main research fields and should provide your availability to assist in their projects. Multicentric studies are usually more scientifically rigorous and, therefore, more likely to be published in high impact journals, and receive more citations.
6. Increase the visibility of your results. In the current situation of an overwhelming number of publications, it is difficult to keep track of the hundreds of scientific manuscripts that are published each week in a specific research area. Recent papers have demonstrated the power of social media in exposing articles to readers which results in a higher number of citations [4,5,6]. Therefore, a good way to positively boost the citations of your paper is to increase its visibility by sharing its main messages through your social media and with colleagues that may be interested in your results.

The above-mentioned tips may help to boost the researcher’s academic profile, thereby leading to a higher number of published papers in high impact journals and citations, and avoiding scientific misconduct. However, there are no universal rules and it is always good to adapt the tips to the researcher’s own practice and choose only the ones that are more likely to fit the specific scientific enviroment of each researcher in order to avoid time-consuming efforts.

Most likely, all these suggestions will improve your scientific productivity and, at the same time, will avoid any temptation of scientific misconduct although the pressure to publish remains high in the academic world.

## Data Availability

Not applicable.
